# Prospective memory and glycemic control in children with type 1 diabetes mellitus: a cross-sectional study

**DOI:** 10.1186/1687-9856-2012-29

**Published:** 2012-12-03

**Authors:** Jennifer N Osipoff, Denise Dixon, Thomas A Wilson, Thomas Preston

**Affiliations:** 1 Division of Pediatric Endocrinology, Department of Pediatrics, Stony Brook Children’s Hospital, HSC Level 11 Room 080, Stony Brook, NY, 11794-8111, USA; 2 Suffolk Health Psychology Services, PLLC, 646 Main Street, 2nd floor Suite 203, Port Jefferson, NY, 11777, USA; 3 Department of Neuropsychology, Stony Brook Medicine, Health Sciences Center, Stony Brook, NY, 11794-8511, USA

**Keywords:** Hemoglobin A1C, Pediatrics, Adolescents, Psychological testing, Diabetes mellitus, Memory

## Abstract

**Background:**

Prospective memory is that memory which is required to carry out intended actions and is therefore essential in carrying out the daily activities required in the self-management of type 1 diabetes mellitus (T1DM). This study aimed to identify the relationships between prospective memory and diabetic control in children with T1DM.

**Method:**

94 children aged 6–18 years with T1DM completed an innovative prospective memory screen, PROMS, and a series of cognitive tests. Parents answered questionnaires about their children's diabetic histories and cognitive skills.

**Results:**

No association between total PROMS score and glycemic control was found. Lower HbA1C was associated with higher (better) scores on the 20 minute event-based task on the PROMS. Parental concerns about working memory and metacognition in their children were mirrored by higher HbA1C.

**Conclusions:**

This study suggests that there may be an association between glycemic control and prospective memory for event based tasks. Additional studies need to be done to determine reproducibility, causality, and if prospective memory based interventions can improve diabetic control.

## Background

Cognitive and executive functioning in type one diabetes mellitus (T1DM) has slowly gained attention in the literature over the past two decades. Studies in this area, particularly within pediatrics, are still scarce. Two recent meta-analyses incorporating studies from 1985–2005 and from 1980–2005 found only 19 and 24 studies respectively that focused on executive function in children with T1DM [1,2]. Both conclude these children have poorer performance on tests of visuo-spatial ability, motor speed, writing, sustained attention, reading, full IQ, performance IQ, and verbal IQ [1,2]. Most of the studies reviewed in these meta-analyses focused on cognition. Research specifically examining pediatric T1DM and memory is even more limited [3-10]. In two reports children with T1DM scored lower on tests of short term memory compared to their healthy peers [4,8]. Hershey *et. al* suggested that severe hypoglycemia had a negative impact on declarative and long-term spatial memory with no such effect seen with procedural memory [3,5-7].

Only two publications have looked at the role that memory plays in diabetic management rather than investigating if T1DM impairs different components of memory [9,10]. Holme*s et. al*. utilized The Wide Range Assessment of Memory and Learning (WRAML), a test designed to assess immediate and delayed recall of verbal and visual memory. This study [9], evaluating youth 9 to 17 years old, concluded that rote verbal memory accounted for 5.5% of the variance in the frequency of blood glucose (BG) testing in children 12.5 years and older and quantitative memory accounted for 9.9% of variance in carbohydrate consumption in adolescents 14.8 years and older. However, these behaviors did not predict mean hemoglobin A1C (HbA1C) values over the prior 6 months. The second paper [10] developed a biopsychosocial model of predictors of youth diabetes care behaviors and metabolic control after studying more than 200 children 9 to 16 years old. A significant inverse correlation of modest magnitude was obtained between higher scores on the memory index of the WRAML and lower HbA1C.

These studies build a foundation for further research involving memory and T1DM. One important gap in our knowledge is the role of prospective memory, an essential component of executing intended actions at an appropriate time in the future, in children with T1DM. Prospective memory is particularly important in T1DM because the medical regimen is complex. Patients must remember to check their BG and count the amount of carbohydrates in foods each time they eat, take insulin a minimum of two times per day, and adjust insulin doses based on their health status, activity, carbohydrate intake and current BG. Adult care takers initially play a large role in this intensive care plan, but as children get older and more independent, more responsibility is placed upon them. Despite its importance in diabetic care, only one study about prospective memory in diabetic subjects has been published. That study looked at prospective memory in adult patients with diabetes under both euglycemic and hypoglycemic conditions and ultimately concluded that acute hypoglycemia impaired prospective memory [11]. The approach used to measure prospective memory in that study was not appropriate for pediatric populations. Research in prospective memory is hindered by the lack of a gold standard for testing. The prospective memory test used in our study, PROMS (see Table 1), was designed with a pediatric population in mind and consists of developmentally appropriate tasks, allowing us to investigate prospective memory in a pediatric T1DM population.

**Table 1:** Description of PROMS

**PROMS Task #**	**Time (min)**	**Instructions to Subject**	**Instructor’s Prompt**	**Subject’s Action**
1	0	1. When I tap on table, get that book for me (EBT)	Gives first two tasks to carry out	
2		2. In exactly two minutes, write your grade in school (TBT)		
3	2	When I show you a manila folder state your phone number (EBT to occur in 5 minutes)		Writes grade in school
4	7	In exactly 5 minutes, get me that CD (TBT)	Shows manila folder	States phone number
5	12	When I show you a stapler tell me to organize my papers (EBT to occur in 10 minutes)		Gets CD
6	20	In exactly 10 minutes put a penny in the envelope (TBT)	Taps on table	Gets book
7	22	When I show you a green paper, write a problem you are having in school (EBT to occur in 15 minutes)	Shows stapler	Says to organize papers
8	30	In exactly 20 minutes ask me what we are going to do next (TBT)		Puts penny in envelope
	37		Shows green paper	Writes down school problem
	50			Asks what are we doing next?

A digital clock is placed on the table between the instructor and the child. The instructor ensures that the child is able to determine what time it will be at different time intervals. Then the instructor gives the child the first two tasks to remember to carry out which starts the test. The instructor gives the next task to remember at the predetermined times regardless of whether or not the child has correctly completed the prior tasks. For scoring purposes tasks are divided into event based tasks (EBT) and time based tasks (TBT).

Our primary study aim was to determine if poorer glycemic control (as measured by higher HbA1C values) is associated with lower prospective memory scores in children with T1DM. Our second aim was to determine if greater parental concerns regarding their child’s cognitive functioning are associated with lower prospective memory scores.

## Methods

One hundred twenty children were recruited during outpatient appointments, hospitalizations, diabetes camp**,** and via advertising in the local Juvenile Diabetes Research Foundation chapter from July 2009 to November 2010. Eligibility criteria were age 6–18 years, diagnosed with T1DM for at least three months, and fluency in English. Exclusion criteria were neurologic, psychiatric, or medical disorders known to effect cognition and attendance in special education programs prior to the diagnosis of T1DM.

The protocol was approved by the institutional review board and the General Clinical Research Center (GCRC); the latter provided grant support (GCRC grant #MO1RR10710) to purchase $40 gift cards for each family to serve as an incentive to participate. Consent and assent were obtained from all parent–child pairs. At the start of the study visit, the child's BG (Accucheck glucometer) and HbA1C (Siemens DCA analyzer) were measured. Hypoglycemia (BG < 70 mg/d), if present, was to be treated prior to testing and the child given a minimum of 20 minutes of recovery time from the hypoglycemic nadir to the onset of memory testing. Cognitive impairment secondary to an acute episode of hypoglycemia has been demonstrated to be fully reversed in this period of time [12]. If the BG was more than 240 mg/dL, urine ketones were checked. No participants had hypoglycemia or moderate or large ketones at the onset of the study.

One GCRC staff member received intensive training from our neuropsychologist (TP) to administer the PROMS and other cognitive tests. This person had no prior interactions with any of the children and remained blinded to the BG and HbA1C results. If hypoglycemia was suspected during testing, the child’s BG was retested and treatment was provided as needed. Only one child experienced low BG during testing.

Prospective memory was assessed using a revised version of the Prospective Memory Screening [13,14] utilizing a combination of eight event-based prospective memory tasks (EBT) and time-based prospective memory tasks (TBT). The PROMS was conducted concurrently with academic testing in order to provide an index of prospective memory that is both sensitive and ecologically valid [15]. The 2-subtest version of the Wechsler Abbreviated Scale of Intelligence (WASI) evaluated general intellect [16]. Academic skills were measured using the Wechsler Individual Achievement Test – Second Edition (WIAT 2) [17]. The California Verbal Learning Test – Children’s Version (CVLT C) assessed declarative memory [18]. Working memory was investigated by the Digit Span subtest of the Wechsler Intelligence Scale for Children – Fourth Edition (WISC 4) [19]. At completion, a repeat BG level was obtained.

The accompanying parent answered questions regarding the child’s diabetic history and the family’s socioeconomic status. Information collected about the child’s diabetic history included age at diagnosis, current insulin regimen, frequency of diabetic ketoacidosis and number of episodes of severe hypoglycemia, defined for our study as loss of consciousness, seizure activity, and/or necessitating glucagon administration. The Behavior Rating Inventory of Executive Functions (BRIEF) was also completed to capture parents’ observations of their child’s capacities across several sub-domains of executive function, including self-regulation, planning/organization, and working memory [20].

### Statistical Analysis

#### Power Analyses

A projected sample size of 100 participants provided 88% power for detecting significant correlations (e.g., for *r* = .30), and a two-tailed significance level set at *p* < .05. The *a priori* power analyses for each regression model (IBM SPSS SamplePower 2.0), included 7 control variables entered in Step 1 of the equation (chronological age at time of PROMS ; age at diabetes diagnosis; age at first severe episode of hypoglycemia; number of episodes of hypoglycemia; duration of T1DM; gender; socioeconomic status), yielding an R-square of .25. The second step of the regression (entering PROMS score) yielded a unique increment of .06 (total R-square for each equation = .31), providing a power of 0.80 for the final increment (and 0.95 for all 8 variables entered into the regression models) with the given sample size of 100 and alpha set at .05. The test was based on Model 2 error, such that variables entered into the regression subsequent to the set of interest served to reduce the error term in the significance test, and therefore were included in the power analysis. This effect was selected as the smallest effect that would be important to detect, in the sense that any smaller effect would not have been of clinical or substantive significance. It was assumed that this effect size was reasonable, in the sense that the effects of this magnitude have been demonstrated by prior research in this field of research [3-6,21,22]. Thus, the analyses provided adequate power for testing the assumptions underlying the statistical models.

#### Data Analysis

All variables were examined for accuracy of data entry, missing values, and fit between their distributions and the assumptions of multivariate analysis. The ratio of cases to independent variables appeared satisfactory for each of the regression analyses. The majority of the variables met the assumptions of normality, linearity, and homoscedasticity of residuals, with the exception of HbA1C and number of severe hypoglycemic episodes. Therefore, these two variables were log transformed prior to entry in the regression analyses.

No significant outliers were identified among the residuals. Co-linearity diagnostics performed for each regression analysis determined that the majority of the independent variables appeared sufficiently independent to enter into the regression analyses, with the exception of age, age at diagnosis and duration of illness, and number of episodes of diabetic ketoacidosis and low BG episodes. As a result, age and number of low BG episodes were selected as empirically based variables for the parsimonious regression equations. The levels of association among the background and control, explanatory, and outcome variables were computed with Pearson product moment correlations. Control variables were entered into hierarchical regression analyses at Step 1, followed by the explanatory variable to determine if the explanatory variable contributed additional unique variance to the multivariate models. Hierarchical regression analyses were deemed appropriate, as they provided primarily descriptive and exploratory information related to cognitive and psychosocial factors as predictors of HbA1C values in a reasonably novel study population. Also, this analysis provided the relative contributions of the delineated independent factors to the outcome variables of HbA1C values. All data were analyzed using IBM SPSS for Mac (Version 18.0).

## Results

One hundred child–parent pairs completed the study. Despite agreeing to participate, twenty families ultimately failed to schedule an appointment with the GCRC. Six children were excluded due to medical co-morbidities or special education needs not disclosed prior to participation. Data from 94 participants was analyzed. Demographic and clinical characteristics are shown in Table 2.

**Table 2:** Demographic and Clinical Characteristics

	**n=94**
Age (years)	12.5 ± 3.4 (range 6.17 -17.95)
Gender (% Female)	49
Ethnicity (% white, not of Hispanic origin)	87.2
Full Scale IQ	106.8 ± 10.5 (range 79–142)
Academic Achievement (%)	
As	53.8
As & Bs	7.7
Bs	28.6
Bs & Cs	5.5
Cs	2.2
Cs & Ds	1.1
As, Bs, & Cs	1.1
Education Level of Mother (%)	
Some High School	1.1
High School Graduate	16
Some College	16
Associate’s Degree	13.8
Bachelor’s Degree	31.9
Master’s Degree	16
Doctorate	3.2
Other	2.1
Education Level of Father (%)	
Some High School	0
High School Graduate	25.5
Some College	19.1
Associate’s Degree	8.5
Bachelor’s Degree	27.7
Master’s Degree	7.4
Doctorate	5.3
Other	6.4
Total household income (%)	
<$25,000	3.2
$25-49,999	7.4
$50-74,999	13.8
$75-99,999	24.5
$100-124,999	23.4
>$125,000	23.4
Didn’t answer	4.3
Caregiver completing study questionnaires (% mother)	81.9
Hemoglobin A1C (%)	7.9 ± 1.2 (range 5.8 – 14.1)
Type 1 diabetes duration (years)	4.9 ± 3.5 (range 0.2 - 12.3)
Age at diagnosis of type 1 diabetes (years)	7.6 ± 3.8 (range 0.9 - 14.9)
Blood glucose at start of study (mg/dL)	209.7 ± 84.5 (range 87 – 452)
Method of Insulin Delivery	
Multiple daily injections (%)	20
CS II (%)	65.6
History of severe low blood glucose reaction (%)	20.4
History of diabetic ketoacidosis (%)	40.4

### Associations of control variables

**Figure 1:**
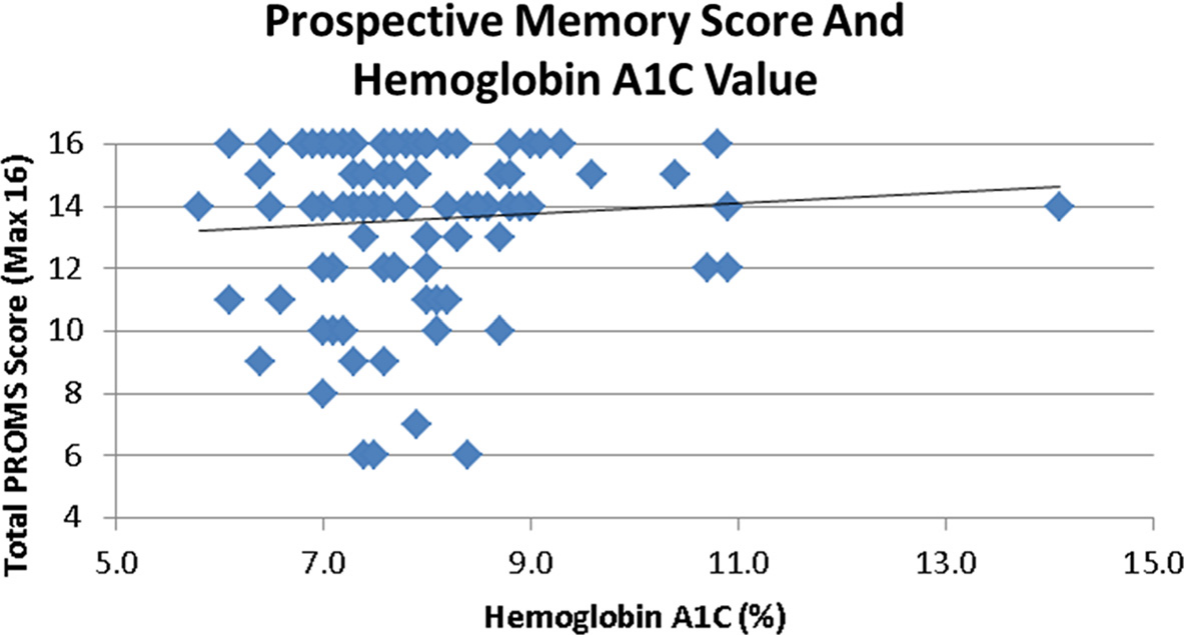
**No correlation was found between glycemic control and total score on the PROMS test**. PROMS scores can range from 0 to 16 points.

No association between total PROMS score and glycemic control was found (see Figure 1). Lower HbA1C values were associated with higher (better) scores on the 20 minute EBT on PROMS (*r* = −0.2, *p* < .05). Parental concerns about their child’s working memory & metacognition were related to higher HbA1C (*r* = 0.21, p < 0.05 for each). No relationship between PROMS scores and parental ratings of the child's cognition on the BRIEF was found. PROMS scores did not correlate with the child’s performance on the standardized cognitive tests, gender, nor BG level at the start of the PROMS.

### PROMS as a predictor of HBA1c values

A hierarchical regression was performed to determine if the PROMS 20 minute EBT would improve predictions of HbA1C values after controlling for participant age, gender, race, socioeconomic status, IQ, and number of severe hypoglycemic episodes. The equation was significant with all of the independent variables entered (*R*^*2*^ = .14, *p* < .05). Increased age predicted higher HbA1C. None of the other control variables were significantly associated with HbA1C values when entered in the same block of the regression equation. The addition of the PROMS 20 minute EBT scores predicted additional variance in HbA1C values, with the beta weight indicating that lower PROMS 20 minute EBT scores predicted higher HbA1C values (β = −0.22, *p* < 0.05). Altogether, 14% (7% adjusted) of HbA1C values were predicted by knowing scores on the independent variable of PROMS 20 minute EBT.

## Discussion

This study represents the first of its kind investigating prospective memory and glycemic control in children with type 1 diabetes mellitus. While as a whole this was a largely negative study, the association between glycemic control and the PROMS 20 minute EBT warrants further exploration. Deficits in EBT could help explain why children, even those with a long standing history of T1DM, have difficulty remembering to check BG and take insulin at meal times, as these events require event-based prospective memory. It is not clear why the only significant correlation between HbA1C and PROMS was the 20 minute EBT and not the shorter EBT. Multiple inputs at the outset of the PROMS and the length of time from request to cue for the task may have contributed to tendency to forget the task. However, this might best mimic expectations outside of the laboratory setting and could impact upon daily diabetic care. Positron emission tomography scans have demonstrated that EBT and TBT activate different areas of the rostral prefrontal cortex [23] and at least one study has shown that deficits in EBT and TBT are not always congruent [24].

Our study also found a modest correlation between HbA1C and parental concerns about the children’s working memory and metacognition as assessed by the BRIEF. This finding is consistent with that reported by McNally *et. al* who concluded that higher levels of executive functioning (i.e. lower reports of parental concern on the BRIEF) was associated with increased treatment adherence which predicted improved HbA1C levels [25].

Most studies involving pediatric T1DM and cognition concluded severely low BG increases the risk of learning difficulties and need for special education [26]; decreased spatial ability and slower motor speeds [6]; impairments in memory, learning, and executive functioning [26]; weaker visual and verbal delayed recall [7,26]; poorer verbal short term memory [2]; inferior analytic skills [21]; and problems with attention [27]. Two studies argue that severe hypoglycemia has no impact on cognition [28,29]. Another concluded that subtle hypoglycemia actually led to an increase in test scores in academic achievement, memory, verbal comprehension and general cognition [30]. Although not one of our primary aims, our study only found a significant correlation between increased hypoglycemic events and increased episodes of diabetic ketoacidosis (*r*= 0.45, p<0.001). While the relationship between increased episodes of hypoglycemia and lower full-scale IQs trended towards significance (*r*= −0.185, p= 0.076), more frequent episodes of severe low BG did not impact performance on the PROMS or academic achievement.

Hyperglycemia, gender, and SES have also been investigated as causal factors of cognitive dysfunction in pediatric T1DM. Conflicting results about the effect of acute hyperglycemia have been published. One study concluded that acute hyperglycemia, defined as >360 mg/dL, had no effect on cognitive functioning [31]. A later study demonstrated that when BG was acutely raised between 360–540 mg/dL in 12 children with T1DM, 8 of the children had a decrease in their performance IQ [32]. Receptive language scores among thirty-six preschoolers with a mean BG of 174mg/dL were found to be inversely correlated with higher blood glucoses; ambient blood glucose did not correlate with other measures of cognitive or motor testing [29]. In the current study, no correlations were seen between ambient BG and PROMS scores, performance on digit span testing, or full scale IQ. Studies testing prospective memory under both euglycemic and hyperglycemic conditions in the same child need to be conducted before conclusions regarding the effect of elevated BG on this component of memory can be made. Several studies have found that boys with T1DM have a higher incidence of learning problems compared to girls with T1DM [33-36]. Our data did not support these findings as gender did not impact prospective memory or cognitive test scores nor did parents of sons report more concerns on the BRIEF. Although lower SES has also been implicated as an additional risk factor for cognitive incongruities [35] this was not found amongst our children.

There are several limitations of this study. While the prospective memory screen used in our study was specifically developed with a pediatric population in mind and has face validity, it is not a standard tool used in psychometric evaluation. However, no other “gold standard” exists. One could argue the shorter tasks measured working memory and not prospective memory, thereby limiting our findings.

The demographic characteristics of our participants were not representative of a broader T1DM population. Our final study population consisted primarily of academically high-achieving children from well-educated, middle to upper class families. As prospective memory is an integral component of daily functioning and success, this self-selected group likely has higher prospective memory skills compared to a more general population of families with children with T1DM. Second, the majority of the participants used intensive insulin regimens and maintained desirable HbA1Cs. It is possible that the prescribing physicians unknowingly assessed aspects of prospective memory skills of these children and their parents and deemed them capable of remembering to carry out the tasks needed for success with an intensive insulin regimen. Third, patients with lower HbA1C, and arguably better prospective memory, may have been more willing to volunteer for the study. Patients with poor glycemic control often failed to come to appointments, theoretically due to deficits in prospective memory, and thus had fewer opportunities to be asked to participate. Generally these patients and their families are seen as “unmotivated” to care for T1DM by health care providers; this lack of motivation may actually represent poor prospective memory skills which make diabetic care that much more challenging for these families.

Mean HbA1C of the 20 consented children who ultimately did not participate in the study despite reminder phone calls and opportunities to reschedule was 9% compared to 7.9% of those who completed the study. As the reminder phone calls were directed to the parents, one must question the adults’ prospective memory and its role in the children’s glycemic control. Future examination of this relationship is warranted, particularly in younger patients in whom adults generally assume responsibility for providing the child’s diabetic care.

The lack of significant relationships between frequency of hypoglycemia and prospective memory, academic achievement, and full-scale IQ may not be generalizable to a more diverse sample of children with higher rates of hypoglycemia. Only 20% of the families reported one or more episode of severe hypoglycemia. The accuracy of this occurrence rate is limited by parental recall and individual interpretation of severe hypoglycemia. The lack of association between lower SES and cognitive functioning may also be due to the homogeneity of our sample, with virtually all of the families earning well above our national poverty threshold.

## Conclusions

Our study introduces the idea that prospective memory and glycemic control may be interrelated in children with T1DM. The association between the 20 minute EBT score and hemoglobin A1C and not the 20 minute TBT raises the possibility that diabetic control only affects event based prospective memory. Further studies need to be conducted to determine causal linkage and direction, as it is equally plausible that either a high HbA1C negatively impacts one’s prospective memory or poor prospective memory predicts difficulty adhering to a diabetic regimen. Additional studies are warranted to determine if deficits in EBT prospective memory are reproducible and to determine how disease duration and fluctuations in HbA1C serially influence this aspect of prospective memory.

## Abbreviations

T1DM: Type One Diabetes Mellitus; HbA1C: Hemoglobin A1C; GCRC: General Clinical Research Center; BG: Blood glucose; BRIEF: Behavior Rating Inventory of Executive Functions; SES: Socio-economic status; EBT: Event based task; TBT: Time based task.

## Competing interests

The authors declare that they have no competing interests.

## Authors’ contributions

JNO conceived of the study, participated in its design, authored the protocol and consent/assent forms submitted for review by the institutional review board, recruited subjects, helped scored the data collected, participated in data analysis, and drafted the manuscript with the exception of the section on statistical analysis. DD participated in the design of the study, completed the statistical analyses, and authored the section on statistical analysis. TAW participated in the study design, edited all material submitted to the institutional review board, recruited subjects, and played a large role in editing the manuscript. TP participated in study design, authored PROMS and trained the GCRC nurse staff in its administration as well as the other cognitive tests, helped in scoring the data collected, and helped draft the manuscript. All authors read and approved the final manuscript.

## References

[B1] Gaudieri PA Chen R Greer TF Holmes CS Cognitive function in children with type 1 diabetes: a meta-analysis Diabetes Care 2008 31 9 1892 1897 10.2337/dc07-2132 18753668 PMC2518367

[B2] Naguib JM Kulinskaya E Lomax CL Garralda ME Neuro-cognitive Performance in Children with Type 1 Diabetes--A Meta-analysis J Pediatr Psychol 2008 34 3 271 282 10.1093/jpepsy/jsn074 18635605

[B3] Hershey T Lillie R Sadler M White NH A prospective study of severe hypoglycemia and long-term spatial memory in children with type 1 diabetes Pediatr Diabetes 2004 5 2 63 71 10.1111/j.1399-543X.2004.00045.x 15189491

[B4] Kovacs M Ryan C Obrosky DS Verbal intellectual and verbal memory performance of youths with childhood-onset insulin-dependent diabetes mellitus J Pediatr Psychol 1994 19 4 475 483 10.1093/jpepsy/19.4.475 7931933

[B5] Hershey T Lillie R Sadler M White NH Severe hypoglycemia and long-term spatial memory in children with type 1 diabetes mellitus: a retrospective study J Int Neuropsychol Soc 2003 9 5 740 750 12901780 10.1017/S1355617703950077

[B6] Hershey T Bhargava N Sadler M White NH Craft S Conventional versus intensive diabetes therapy in children with type 1 diabetes: effects on memory and motor speed Diabetes Care 1999 22 8 1318 1324 10.2337/diacare.22.8.1318 10480777

[B7] Hershey T Craft S Bhargava N White NH Memory and insulin dependent diabetes mellitus (IDDM): Effects of childhood onset and severe hypoglycemia J Int Neuropsychol Soc 1997 3 509 520 9448364

[B8] Wolters CA Yu SL Hagen JW Kail R Short-term memory and strategy use in children with insulin-dependent diabetes mellitus J Consult Clin Psychol 1996 64 6 1397 1405 8991326 10.1037//0022-006x.64.6.1397

[B9] Soutor SA Chen R Streisand R Kaplowitz P Holmes CS Memory matters: developmental differences in predictors of diabetes care behaviors J Pediatr Psychol 2004 29 7 493 505 10.1093/jpepsy/jsh052 15347698

[B10] Holmes CS Chen R Streisand R Marschall DE Souter S Swift EE Peterson CC Predictors of youth diabetes care behaviors and metabolic control: a structural equation modeling approach J Pediatr Psychol 2006 31 8 770 784 16221954 10.1093/jpepsy/jsj083

[B11] Warren RE Zammitt NN Deary IJ Frier BM The effects of acute hypoglycaemia on memory acquisition and recall and prospective memory in type 1 diabetes Diabetologia 2007 50 1 178 185 17143604 10.1007/s00125-006-0535-6

[B12] Evans ML Pernet A Lomas J Jones J Amiel SA Delay in onset of awareness of acute hypoglycemia and of restoration of cognitive performance during recovery Diabetes Care 2000 23 7 893 897 10.2337/diacare.23.7.893 10895837

[B13] Sohlberg MM Mateer CA Geyer S Prospective Memory Survey 1985 Puyallup: Assocation for Neuropsychological Research and Development

[B14] Sohlberg M Mateer C Geyer S Preston T Prospective Memory Screening Test- Revised Unpublished Test Manual 2009 Stony Brook, NY: Developed at Stony Brook University Medical Center

[B15] Preston T Eisenberg P Prospective Memory in Clinically Referred and Nonreferred Children 2011 Boston, MA: Presented at 39th Annual Meeting of the International Neuropsychological Society

[B16] Wechsler D Wechsler Abbreviated Scale of Intelligence (WASI) 1999 San Antonio Texas: Psychological Corporation

[B17] Harcourt Assessment, Inc Wechsler Individual Achievement Test- Second Edition (WIAT 2) 2002 San Antonio Texas: Psychological Corporation

[B18] Delis D Kramer JH Kaplan E Ober BA California Verbal Learning Test- Children's Version (CVLT-C) 1994 San Antonio, Texas: Psychological Corporation

[B19] Wechsler D Wechsler Intelligence Scale for Children- Fourth Edition (WISC 4) 2004 San Antonio, Texas: Psychological Corporation

[B20] Gioia GA Isquith PK Guy SC Kenworthy L Behavior Rating Inventory of Executive Function 2000 Lutz, Florida: Psychological Assessment Resources

[B21] Perantie DC Lim A Wu J Weaver P Warren SL Sadler M White NH Hershey T Effects of prior hypoglycemia and hyperglycemia on cognition in children with type 1 diabetes mellitus Pediatr Diabetes 2008 9 2 87 95 10.1111/j.1399-5448.2007.00274.x 18208449

[B22] Kovacs M Goldston D Iyengar S Intellectual Development and Academic Performance of Children with Insulin-Dependent Diabetes Mellitus: A Longitudinal Study Dev Psychol 1992 28 4 676 684

[B23] Okuda J Fujii T Ohtake H Tsukiura T Yamadori A Frith CD Burgess PW Differential involvement of regions of rostral prefrontal cortex (Brodmann area 10) in time- and event-based prospective memory Int J Psychophysiol 2007 64 3 233 246 10.1016/j.ijpsycho.2006.09.009 17126435

[B24] Katai S Maruyama T Hashimoto T Ikeda S Event based and time based prospective memory in Parkinson's disease J Neurol Neurosurg Psychiatry 2003 74 6 704 709 10.1136/jnnp.74.6.704 12754335 PMC1738496

[B25] McNally K Rohan J Pendley JS Delamater A Drotar D Executive functioning, treatment adherence, and glycemic control in children with type 1 diabetes Diabetes Care 2010 33 6 1159 1162 10.2337/dc09-2116 20215458 PMC2875415

[B26] Hannonen R Tupola S Ahonen T Riikonen R Neurocognitive functioning in children with type-1 diabetes with and without episodes of severe hypoglycaemia Dev Med Child Neurol 2003 45 4 262 268 12647928 10.1017/s0012162203000501

[B27] Ryan C Vega A Drash A Cognitive deficits in adolescents who developed diabetes early in life Pediatrics 1985 75 5 921 927 3991281

[B28] Wysocki T Harris MA Mauras N Fox L Taylor A Jackson SC White NH Absence of adverse effects of severe hypoglycemia on cognitive function in school-aged children with diabetes over 18 months Diabetes Care 2003 26 4 1100 1105 10.2337/diacare.26.4.1100 12663580

[B29] Patino-Fernandez A Delamater AM Applegate EB Brady E Eidson M Nemery R Gonzalez-Mendoza L Richton S Neurocognitive Functioning in Preschool-age Children with Type 1 Diabetes Mellitus Pediatr Diabetes 2010 11 6 424 430 10.1111/j.1399-5448.2009.00618.x 20456084 PMC2921563

[B30] Kaufman FR Epport K Engilman R Halvorson M Neurocognitive functioning in children diagnosed with diabetes before age 10 years J Diabetes Complications 1999 13 1 31 38 10.1016/S1056-8727(98)00029-4 10232707

[B31] Gschwend S Ryan C Atchison J Arslanian S Becker D Effects of acute hyperglycemia on mental efficiency and counterregulatory hormones in adolescents with insulin-dependent diabetes mellitus J Pediatr 1995 126 2 178 184 10.1016/S0022-3476(95)70542-2 7844662

[B32] Davis EA Soong SA Byrne GC Jones TW Acute hyperglycaemia impairs cognitive function in children with IDDM J Pediatr Endocrinol Metab 1996 9 4 455 461 8910814 10.1515/jpem.1996.9.4.455

[B33] Fox MA Chen RS Holmes CS Gender differences in memory and learning in children with insulin-dependent diabetes mellitus (IDDM) over a 4-year follow-up interval J Pediatr Psychol 2003 28 8 569 578 10.1093/jpepsy/jsg047 14602847

[B34] Holmes CS Dunlap WP Chen RS Cornwell JM Gender differences in the learning status of diabetic children J Consult Clin Psychol 1992 60 5 698 704 1401385 10.1037//0022-006x.60.5.698

[B35] Holmes CS Cant MC Fox MA Lampert NL Greer T Disease and Demorgraphic Risk Factors for Disrupted Cognitive Functioning in Children with Insulin-Dependent Diabetes Mellitus Sch Psychol Rev 1999 28 215 227

[B36] Holmes CS O'Brien B Greer T Cognitive functioning and academic achievement in children with insulin-dependent diabetes mellitus (IDDM) Prof Sch Psychol 1995 10 4 329 344

